# A Two-State Model for the Dynamics of the Pyrophosphate Ion Release in Bacterial RNA Polymerase

**DOI:** 10.1371/journal.pcbi.1003020

**Published:** 2013-04-04

**Authors:** Lin-Tai Da, Fátima Pardo Avila, Dong Wang, Xuhui Huang

**Affiliations:** 1 Department of Chemistry, The Hong Kong University of Science and Technology, Clear Water Bay, Kowloon, Hong Kong; 2 Skaggs School of Pharmacy and Pharmaceutical Sciences, University of California San Diego, La Jolla, California, United States of America; 3 Division of Biomedical Engineering, The Hong Kong University of Science and Technology, Clear Water Bay, Kowloon, Hong Kong; 4 Center of Systems Biology and Human Health, Institute for Advance Study and School of Science, The Hong Kong University of Science and Technology, Clear Water Bay, Kowloon, Hong Kong; Ottawa University, Canada

## Abstract

The dynamics of the PP_i_ release during the transcription elongation of bacterial RNA polymerase and its effects on the Trigger Loop (TL) opening motion are still elusive. Here, we built a Markov State Model (MSM) from extensive all-atom molecular dynamics (MD) simulations to investigate the mechanism of the PP_i_ release. Our MSM has identified a simple two-state mechanism for the PP_i_ release instead of a more complex four-state mechanism observed in RNA polymerase II (Pol II). We observed that the PP_i_ release in bacterial RNA polymerase occurs at sub-microsecond timescale, which is ∼3-fold faster than that in Pol II. After escaping from the active site, the (Mg-PP_i_)^2−^ group passes through a single elongated metastable region where several positively charged residues on the secondary channel provide favorable interactions. Surprisingly, we found that the PP_i_ release is not coupled with the TL unfolding but correlates tightly with the side-chain rotation of the TL residue R1239. Our work sheds light on the dynamics underlying the transcription elongation of the bacterial RNA polymerase.

## Author Summary

Pyrophosphate ion (PP_i_) release is a critical step in the nucleotide addition cycle of transcription elongation. Despite extensive experimental studies, the kinetic mechanism of the PP_i_ release in bacterial RNA polymerases (RNAP) still remains largely a mystery. As a cellular machine, RNAP contains more than 3000 residues, and thus it is challenging for all-atom molecular dynamics (MD) simulations to directly capture the process of the PP_i_ release. In this study, we have simulated the dynamics of the PP_i_ release at microsecond timescale using the Markov State Models (MSMs) built from extensive MD simulations in explicit solvent. MSM is a powerful kinetic network model and can predict long timescale dynamics from many short MD simulations. Our results suggest a simple two-state model for the PP_i_ release in RNAP, which sharply contrasts with the more complex four-state hopping model in the yeast RNA polymerase (Pol II). We also observe a 3-fold faster dynamics for the PP_i_ release in RNAP compared to Pol II, consistent with the faster transcription rate in the bacterial systems. Our results greatly improve our understanding of the PP_i_ release, and also provide predictions to guide future experimental tests.

## Introduction

The DNA-dependent RNA polymerase is the main enzyme that participates in the transcription process transferring the genetic information from DNA to messenger RNA (mRNA) [1]. Crystallographic structures of the multi-subunit RNA polymerases in eukaryotes [2]–[4] and bacteria [5]–[8] engaged in transcription elongation process have been obtained. These atomic-level structures provide static snapshots of the transcription cycle [9]–[14].

In each nucleotide addition cycle (NAC) of the multi-subunit RNA polymerase, the post-translocation state first allows the substrate NTP to bind to the active site [6]. Then, a critical domain, named trigger loop (TL), can fold then expel the solvent from the active site [15]–[17], and finally form direct contacts with the substrate NTP. Substitution of a conserved TL histidine can significantly decrease the polymerization rate [18]–[21]. Recent mutagenesis studies have shed light on the roles of the TL on the nucleotidyl transfer [20], [21], and the reverse intrinsic hydrolysis process [22]. Previous MD simulation studies also provided information on TL dynamics and its potential regulatory roles during the translocation process [23], [24]. After the catalytic reaction, PP_i_ forms and releases from the active site [25], [26]; then the TL opens and allows the template DNA to translocate so that a new NAC can start. Extensive biochemical and theoretical studies have been performed to understand the specific steps in the NAC, such as motions of the TL [17], catalysis [26]–[30], translocation [23], [24], [31]–[33] and NTP binding [34], [35].

PP_i_ release in single subunit T7 RNA polymerase is proposed to be tightly coupled with the translocation [36] but the same coupling is not observed in *Escherichia coli* (*E. coli*) RNA polymerase [37]. Interestingly, recent fluorescence and biochemical studies found that the PP_i_ release in the *E. coli* RNA polymerase occurs shortly before or concurrently with the translocation [33]. Nonetheless, the interplay between the PP_i_ release step and the TL opening motions at molecular level is still elusive. Previously, we used MD simulations to study the PP_i_ release in the eukaryotic RNA polymerase II (Pol II) [25]. We proposed a hopping model for Pol II in which PP_i_ release was coupled with the TL tip motion through the interactions between the TL residue H1085 and the (Mg-PP_i_)^2−^ group, and subsequently hopping among several positive charged residues in the secondary channel. Our model further suggested that the PP_i_ release is a fast dynamic process so that it may not be able to induce the fully TL opening motion.

**Figure 1:**
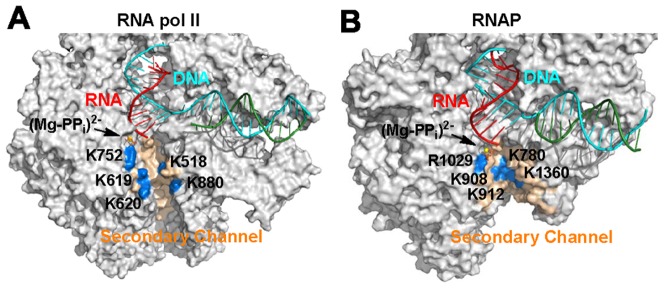
Comparison of the secondary channel (in wheat) of RNA Pol II (A) and RNAP (B). For both structures, RNA, template DNA and non-template DNA are shown in red, cyan and green, respectively. The (Mg-PP_i_)^2−^ group is represented in stick and sphere models. Several critical residues in the channel: K752, K619, K620, K518 and K880 in RNA Pol II; R1029, K908, K912, K780 and K1360 in RNAP, are highlighted in blue. The Pol II model used to make this figure was taken from our previous study [25].

A comparison of the secondary channel and TL structure between Pol II and bacterial RNA polymerase (RNAP) from *T. thermophilus* (*Tth*) displays substantial differences (See Figure 1) [3], [7]. In Pol II, the TL contains a long loop domain (from the Rpb1 residue T1080 to T1095) [3]. However, the TL in RNAP consists of two alpha helices connected by a short turn in the closed state [6]. This structural difference suggests that the dynamics of the TL folding in these two systems are likely to be different. Moreover, in addition to the conserved *Tth* TL residue H1242, the *Tth* TL residue R1239 also interacts with the substrate NTP [6]; this residue is absent in Pol II and mutation of the counterpart residue in *E. coli* (R933A) can reduce the nucleotide addition rate [20]. Moreover, the secondary channel in *Tth* RNAP is much shorter than that in Pol II (See Figure 1), and exhibits a different layout of the positively charged residues. Specifically, in Pol II, the four residues, K619, K620, K518 and K880 are located at relatively separated sites (See Figure 1A). However, the positively charged residues in *Tth* RNAP: K908, K912, K780 and K1369 are close to each other in a continuous region (See Figure 1B). Given these structural differences, it is of interest to compare the dynamics of PP_i_ release in RNAP with that in Pol II.

Although conventional all-atom MD simulations can provide the dynamic information for biological macromolecules at atomic resolution, it is still challenging to capture the biologically relevant timescales in microseconds or even longer. Markov State Models (MSMs) constructed from a large number of short simulations provide one way to overcome this timescale gap [38], [39]. MSMs have been successfully applied to model the long timescale dynamics that cannot be directly accessed by conventional MD simulations in studying the conformational changes of biological macromolecules [39]–[41], including our previous study of PP_i_ release in Pol II [25].

In this study, in order to reveal the mechanism of the PP_i_ release in RNAP, we constructed a MSM from extensive all-atom molecular dynamics (MD) simulations in explicit solvent with a system size of nearly 300,000 atoms and aggregated simulation time of ∼1 µs. Our results reveal that the PP_i_ release in *Tth* RNAP adopts a simple two-state model with a fast dynamics over a few hundred nanoseconds. Surprisingly, we found that the PP_i_ release is not coupled with the secondary structure unfolding of TL but only with the side-chain rotation of the TL residue R1239.

## Results

### PP_i_ release in bacterial RNAP follows a simple two-state model

To study the release mechanism of the (Mg-PP_i_)^2−^ group in RNAP, we modeled the PP_i_-bound RNAP complex by directly cleaving the P_α_-O bond in the ATP-bound RNAP complex that is derived from the *Tth* RNAP crystal structure (See SI Figure S1 for the two structures and the Methods section for the modeling details) [6]. This modeled PP_i_-RNAP complex was used as the starting structure for the steered MD (SMD) simulations to obtain the initial release pathways. To eliminate the bias in SMD simulations, we have then performed 100 10-ns MD simulations, and these simulations have widely sampled the region in the secondary channel (See SI Figure S2). Finally, we have constructed a MSM from these simulations to obtain the dynamics and other thermodynamic properties of the PP_i_ release (See the Methods section for details).

**Figure 2:**
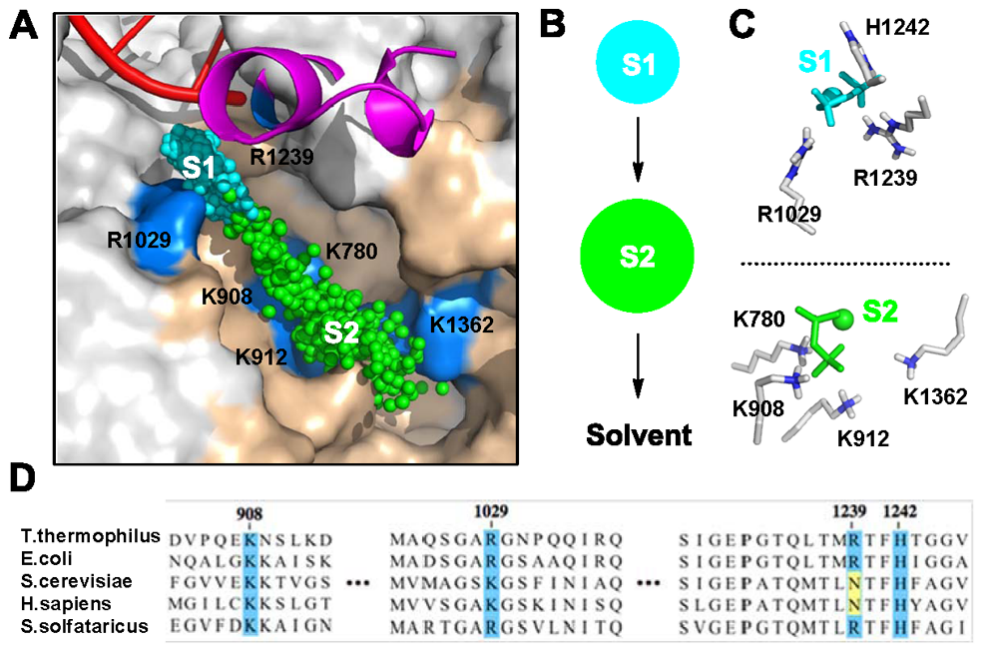
A two-state mechanism for the PP_i_ release in RNAP revealed by the MSM. (A) Two metastable states (S1 and S2) are identified. 500 randomly selected conformations from each metastable state are superimposed and represented with cyan and green spheres for S1 and S2 respectively. Each sphere indicates the coordinate of the center of mass of the PP_i_ group. (B) The two metastable states are displayed as two circles, and the size of these circles is proportional to the equilibrium populations of the S1 (12.6%±0.02%) and S2 (87.4%±0.02%) state, (C) Key interactions between (Mg-PP_i_)^2−^ group and RNAP in each state are displayed. (D) Conservation analysis of the positively charged residues that interact with the (Mg-PP_i_)^2−^ group among different species. The sequence alignment was performed using the online software ClustalW2 (http://www.ebi.ac.uk/Tools/msa/clustalw2/).

Our MSM shows that the PP_i_ release in *Tth* RNAP adopts a simple two-state model. In addition to the initial state with the PP_i_ in the active site (S1 state in Figure 2A), only one additional metastable state is identified (S2 state in Figure 2A), and this state is ∼7-fold more populated than the S1 state (See Figure 2B). The S2 state locates in an elongated region where several positively charged residues can stabilize the (Mg-PP_i_)^2−^ group. These results contrast with our previous findings that the (Mg-PP_i_)^2−^ group in Pol II hops through four clearly separated metastable states [25].

**Figure 3:**
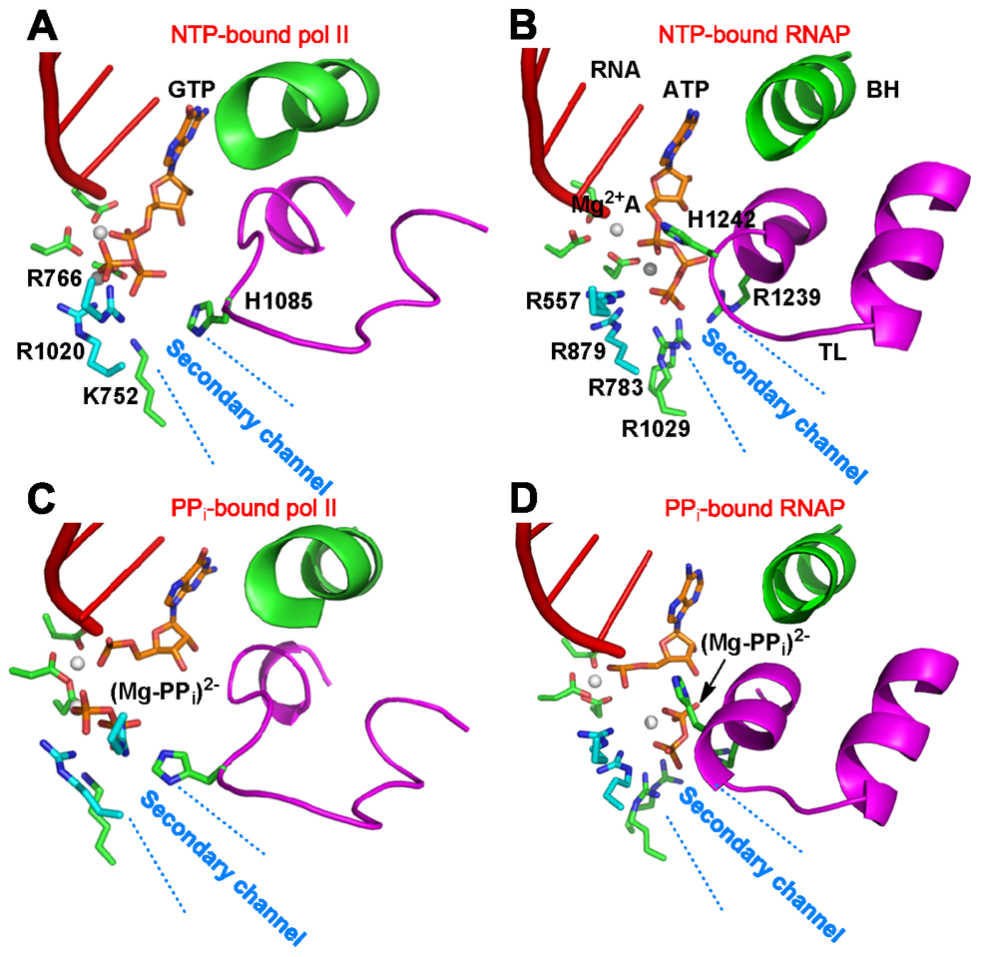
Different binding modes between the TL histidine in Pol II (H1085) and RNAP (H1242) with the (Mg-PP_i_)^2−^ group. (A) and (B) are the structures of the NTP-bound RNA Pol II and RNAP complexes respectively. (C) and (D) are the corresponding PP_i_-bound models. The Bridge Helix (BH, in green), Trigger Loop (TL, in magenta), RNA chain (in red), NTP or PP_i_ (orange and blue), Mg^2+^ atoms (in white), and selected residues in the active sites are displayed.

When the (Mg-PP_i_)^2−^ group is in the active site (See Figure 2C), three positively charged β′ residues R1029, H1242 and R1239 can interact with the negatively charged (Mg-PP_i_)^2−^ group. The residue R1029 locates at the exit of the active site, and thus it may play similar roles on the PP_i_ release with its corresponding residue K752 in Pol II (See Figure 2D) [25]. Interestingly, the location of the conserved TL residue H1242 is different from its counterpart residue H1085 in Pol II, though both of them are in direct contact with the (Mg-PP_i_)^2−^ group. Both before and after chemistry, H1242 interacts with the P_α_-O atom of the NTP in RNAP, whereas H1085 is in contact with P_β_-O atom in Pol II (See Figure 3) [3], [6]. To achieve this, the H1242 in RNAP has to locate deeper in the active site compared to H1085. Finally, R1239 in RNAP locates at the same position as H1085 in Pol II, suggesting that these two residues may play similar roles in the PP_i_ release.

After escaping from the active site, the (Mg-PP_i_)^2−^ group reaches the S2 state with an elongated shape. In this state, multiple positively charged residues on the secondary channel (K780, K908, K912 and K1362) can provide favorable electrostatic interactions with the negatively charged (Mg-PP_i_)^2−^ group (See Figure 2C). In contrast, the (Mg-PP_i_)^2−^ group in Pol II is found to transfer through several hopping sites where groups of positively charged residues are spatially well separated (See Figure 1A) [25]. From the S2 state, the (Mg-PP_i_)^2−^ group will directly enter the solvent. In order to elucidate the specific roles of the three important residues: R1029, H1242 and R1239 in the PP_i_ release (See Figure 2D), we performed additional mutant simulations starting from several different conformations from the S1 and S2 states.

### Specific roles of several positively charged residues in PP_i_ release revealed by mutant simulations

**Figure 4:**
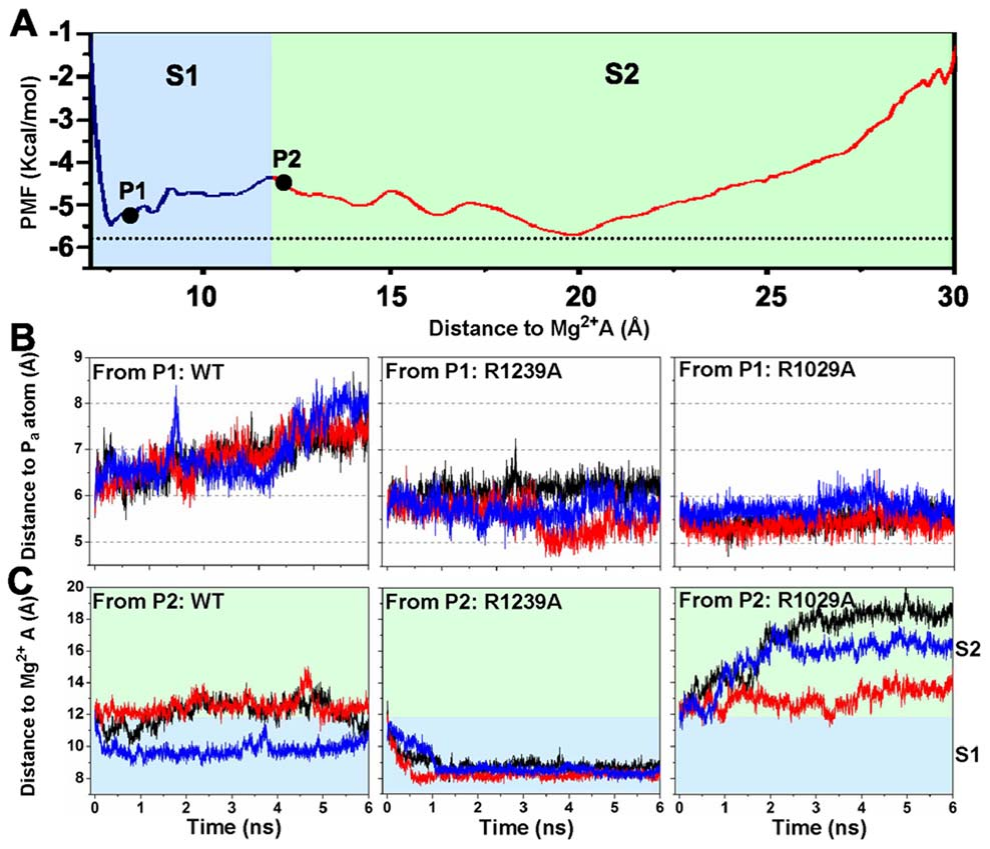
Single mutant simulations reveal the roles of the critical residues H1242, R1239 and R1029 in the PP_i_ release. (A) Potential of mean force (PMF) plot along the distance between the PP_i_ group and Mg^2+^A. The initial conformations of the mutant simulations are highlighted as black spheres (P1 and P2). (B) The distance between the PP_i_ group and the P_α_atom as a function of the simulation time for WT (left panel), R1239A (middle panel), and R1029A (right panel) simulations initiated from P1. We chose this reaction coordination because this distance can directly measure the relative motions between the terminal RNA nucleotide and the PP_i_ group before it leaves the active site. (C) The same as (B) except that all the simulations were initiated from P2, and the distance between the PP_i_ group and the Mg^2+^A was shown. In (A) and (C), the S1 and S2 state are highlighted in blue and light green respectively.

The potential of mean force (PMF) profile along the distance between the (Mg-PP_i_)^2−^ group and the Mg^2+^A is displayed in Figure 4A. The PMF plot shows two major free energy basins that are consistent with the two metastable states identified by our MSM. The starting structures chosen for the mutant simulations fall into two different regions in the PMF profile (P1 and P2 sites in Figure 4A). The P1 site is located in the S1 state, while the P2 site is located in the S2 state but near to the boundary between the S1 and S2 states. Initial conformations from these two sites allow us to examine the roles of the residues involved in different stages of the PP_i_ release.

The mutant simulation results indicate that both residues R1239 and R1029 can facilitate the escape of the (Mg-PP_i_)^2−^ group from the active site to S1 state (See Figure 4B). Here, we use the distance between the (Mg-PP_i_)^2−^ group and the P_α_ atom of the 3′-terminal nucleotide of the RNA chain (*d*_αβ_) to describe the extent of the PP_i_ release from the active site. In the WT simulations (See P1 in Figure 4A), the (Mg-PP_i_)^2−^ group can move towards the exit of active site with the *d*_αβ_ value increasing from 6 Å to around 8 Å (See the left panel of Figure 4B). However, the R1239A and R1029A mutants lead to a weaker tendency for the (Mg-PP_i_)^2−^ group to escape the active site (the *d*_αβ_ value fluctuates around 5.5 Å, middle and right panels in Figure 4B). On the other hand, the R1029K mutant is shown to have a similar effect to help the (Mg-PP_i_)^2−^ group to leave the active site as in WT(see Figure S4A). These results indicate that positively charged residues play a crucial role to facilitate the PP_i_ to release from the active site.

Notably, the H1242A mutant can dramatically promote the PP_i_ release from P1 site (See SI Figure S4C), suggesting that H1242 may prevent the PP_i_ release from the active site. In contrast, the TL residue H1085 in Pol II was previously found to facilitate the PP_i_ release from the active site [25]. This difference may be due to the different locations of these two residues in the active site. Compared with H1085 in Pol II, H1242 in RNAP locates significantly deeper inside of the active site (See Figure 3D). Therefore, it will be more difficult for H1242 to rotate and help the (Mg-PP_i_)^2−^ group to leave the active site. Instead, H1242 can provide an attractive interaction to prevent the PP_i_ release.

Next, we evaluated the roles of residues R1239 and R1029 in PP_i_ release when the (Mg-PP_i_)^2−^ group is at the S2 state (P2 in Figure 4A). In the WT system, the (Mg-PP_i_)^2−^ group fluctuates around its initial location within our simulations at a few nanoseconds, which was also observed in the R1029K mutant simulations initiated from P2 (Figure 4C). Intriguingly, R1029A and R1239A substitutions lead to dramatic, but opposite, effects. The R1029A substitution facilitates the PP_i_ release toward the solvent (Figure 4C, right panel). Combined with the previous observations, we conclude that the R1029 may facilitate the PP_i_ release from the active site but prevents the PP_i_ release when it arrives at the S2 state. Thus R1029 plays a similar role as the corresponding residue K752 in Pol II (See Figure 2D) [25]. In contrast, the R1239A substitution drives the (Mg-PP_i_)^2−^ group back to the S1 state, suggesting that R1239 is critical for the (Mg-PP_i_)^2−^ group to escape the active site (middle panel in Figure 4C). This indicates that R1239, rather than H1242 residue, plays the role in PP_i_ release most equivalent to that played by H1085 in Pol II. Compared with its counterpart residue H1085 in Pol II, the R1029 has a longer and more flexible side chain. In addition, it can form a stronger salt bridge with the (Mg-PP_i_)^2−^ group. Therefore, the side-chain rotation of R1239 alone may be sufficient to facilitate the PP_i_ release.

#### PP_i_ release does not induce the TL backbone unfolding, but is tightly coupled with the side-chain rotation of the TL residue R1239

In order to reveal if the PP_i_ release is coupled with the TL unfolding in RNAP, we have monitored the structural changes of the TL during the PP_i_ release. We calculated the RMSD values of the heavy atoms (non-hydrogen) for both the complete TL domain (β′ residue Q1235 to G1255) and its tip part (residues R1239 to A1249). RMSD values for the complete TL mostly fluctuate between 1 and 3 Å (See SI Figure S5A), indicating that TL does not unfold during the PP_i_ release. On the other hand, for the tip part of the TL, the RMSD values mostly fluctuate between 1 and 2.5 Å when the (Mg-PP_i_)^2−^ group is in the S1 state (See SI Figure S5B). But RMSD values increase to between 2 and 4.5 Å when the (Mg-PP_i_)^2−^ group reaches the S2 state, indicating that the tip region of the TL becomes more flexible after the (Mg-PP_i_)^2−^ group leaves the active site. The Mean First Passage Time (MFPT, the average transition time from S1 state to S2 state) for the PP_i_ release is around 0.5 µs (See SI Table S1 and the Methods section for MFPT calculation details), which is three-fold faster than that in Pol II (∼1.5 µs) [25]. These results further support the idea that PP_i_ release occurs too fast to lead directly to unfolding of the TL helices.

**Figure 5:**
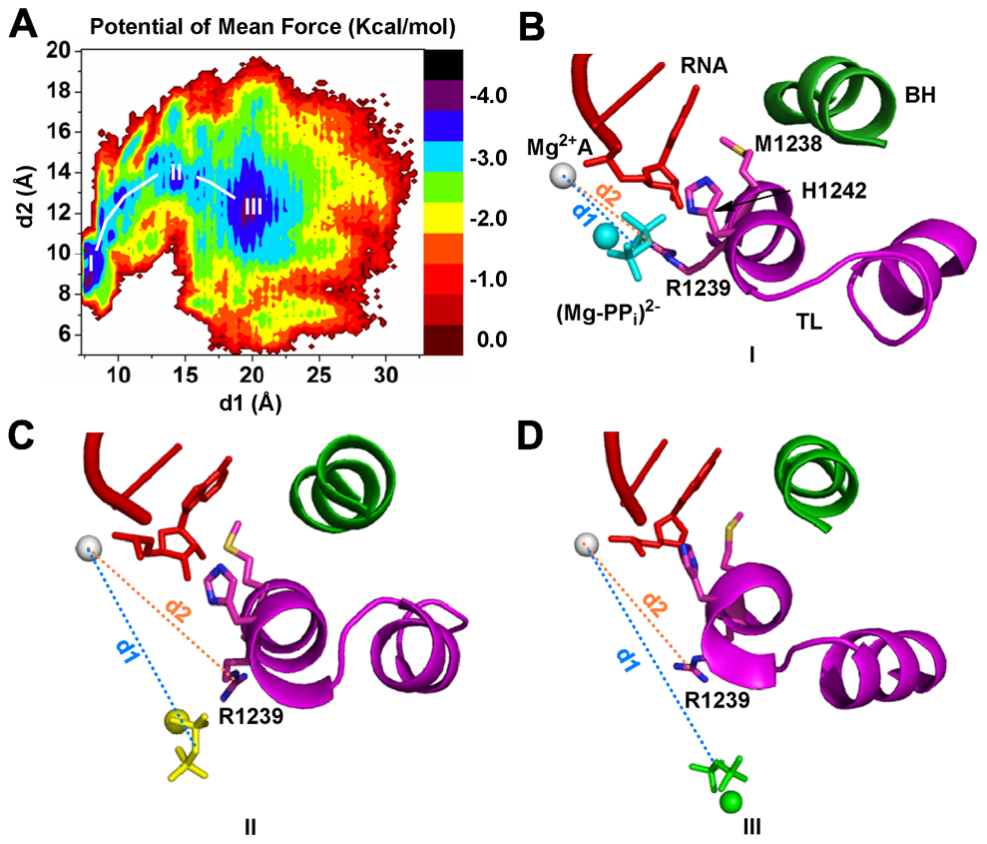
Side chain rotation of the TL residue R1239 facilitates the PP_i_ release. (A) Potential of mean force (PMF) of the (Mg-PP_i_)^2−^ conformations projected on two reaction coordinates: d_1_ (distance between the PP_i_ group and Mg^2+^A) and d_2_ (distance between the Guanidine C atom from the R1239 and Mg^2+^A). Representative structures from three free energy minima labeled as I, II and III in the PMF plot are shown in (B), (C) and (D) respectively. The free energy minimum I belongs to the S1 state, while the other two belong to the S2 state. The structural presentation is the same as Figure 3.

More interestingly, we found a direct correlation between the PP_i_ release and the side chain rotation of the TL residue R1239. The PMF profile in Figure 5A clearly shows that the transition of the (Mg-PP_i_)^2−^ group from the S1 to S2 state correlates with the rotational motion of the residue R1239, with its distance to the Mg^2+^A ion increasing from 10 to 14 Å (See Figure 5B and C). These results are consistent with our previous observation that R1239 can facilitate the PP_i_ release. When the (Mg-PP_i_)^2−^ group arrives at the S2 state (See Figure 5D), its interaction with the residue R1239 is lost, and this increase the fluctuations of the residue R1239 (See Figure 5A).

We did not observe the backbone unfolding of the TL during the PP_i_ release in our model. However, since the timescale for the PP_i_ release is an order of magnitude longer than our individual seeding MD simulations, there exists a possibility that the seeding MD simulations may be biased by initial conformations obtained from steered MD, where the TL is always folded. We thus performed control simulations of both isolated TL domain and a truncated model in which all the motifs surrounding the TL domains were included to further investigate its folding. For an isolated TL domain (from A1225 to A1265) in free solution, it can quickly unfold at ∼210-ns (See SI Figure S6A–C). However, the inclusion of the RNAP motifs surrounding the TL domain can shield not only TL helix facing the active site but also part of the other TL helix facing the secondary channel from the solvent. This may greatly stabilize the secondary structure of the TL domain and prevent it from unfolding. Indeed, no unfolding events were observed in two independent 300-ns MD simulations, and the secondary structure of the TL domain was well preserved (See SI Figure S7A–C). These control simulations suggest that the TL domain must be exposed to the solvent before its secondary structures can be unfolded. We speculate that the side chain rotation of the TL residue R1239 may initiate and allow the overall motion of the TL domain, and this will in turn make the TL more exposed to the solvent and eventually unfold. Interestingly, one recent crystal structure of the RNAP captures an open state of the TL, and in this structure the completely solvent-exposed segment (TL residues from 1246 to 1254) is unfolded but, the segment that is not fully exposed to the solvent remains folded (TL residues from 1235 to 1243) [7]. Based on the control simulations, we conclude that the full opening motion of the TL likely occurs at a timescale longer than the timescale of PP_i_ release.

## Discussion

**Figure 6:**
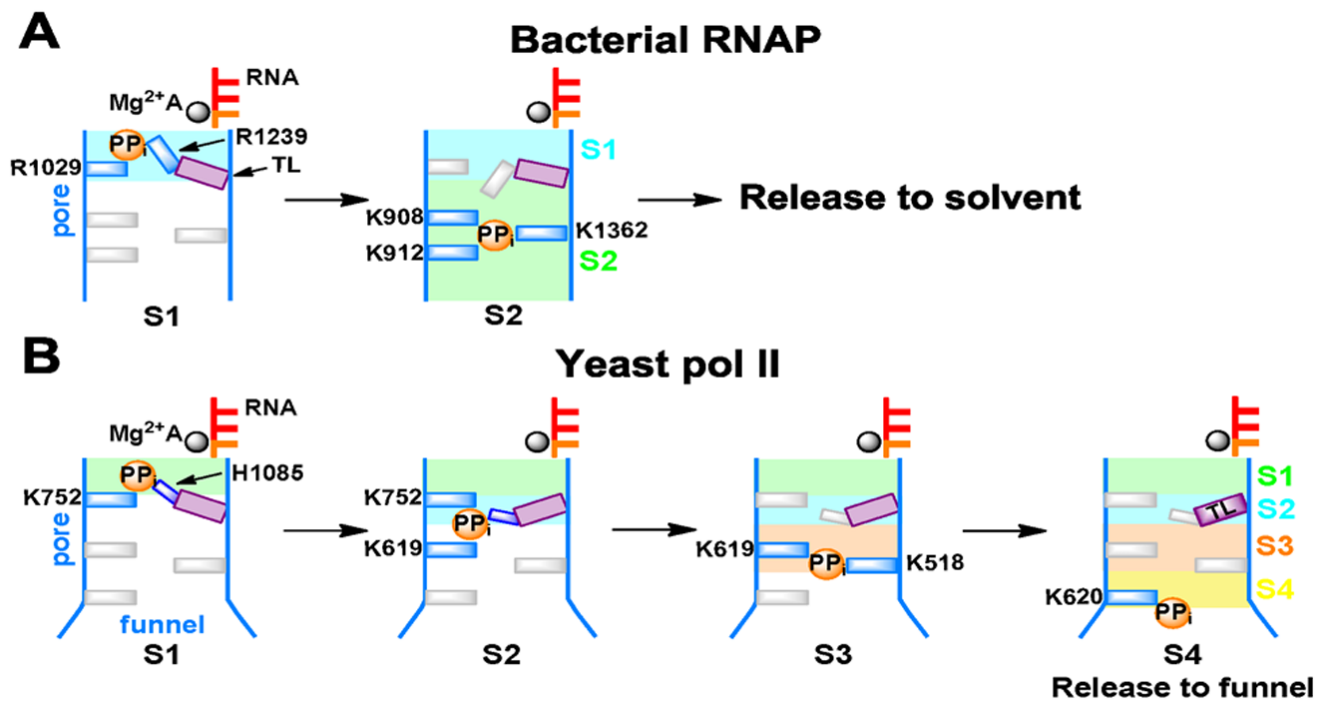
Cartoon models of the PP_i_ release in RNAP (A) and Pol II (B).

Based on extensive unbiased MD simulations, we built a MSM for the PP_i_ release in RNAP to elucidate its long timescale dynamics. The MSM identified a two-state model for the PP_i_ release (See Figure 6A). The mutant simulations indicate that the β′ residues R1239 and R1029 can facilitate the escape of the (Mg-PP_i_)^2−^ group from the active site after the catalytic reaction (See Figure 4B). Then the (Mg-PP_i_)^2−^ group transfers to the S2 state, where it forms favorable interactions with four positively charged residues on the secondary channel: K908, K912, K780 and K1369 (See Figure 6A). More strikingly, our work suggests that the PP_i_ release does not induce the TL unfolding but tightly couples to the side-chain rotation of the TL residue R1239, which in turn makes the TL tip region more flexible. Furthermore, our control simulations show that TL is stable in Pol II, but can quickly unfold (within 200 ns) when exposed to the solvent. We thus speculate that the rotation of R1239 that accompanies the PP_i_ release may allow solvent to re-enter the active site and promote the overall movements of the TL domain; This TL movements would further lead to its exposure to solvent and eventually allow TL unfolding. However, the timescales for this solvent-induced TL unfolding may be significantly longer than that of the PP_i_ release so that we didn't observe it in our simulations.

We found that the TL in RNAP may be more difficult to unfold than that of Pol II, since its secondary structures barely unfold upon the PP_i_ release. Therefore, if the open state of the TL is a pre-requisite step for the translocation as recently suggested by both experimental [33] and computational studies [24], it is intriguing that the transcription rate for bacterial RNAP is much faster than that of Pol II [27]. Despite that the more stable secondary structure of the TL in RNAP may slow down its opening motion, its reverse closing motion may be spontaneous and fast. This fast closing motion may further accelerate the nucleotide addition process to achieve an overall fast transcription rate.

Finally, our MSM indicates that the PP_i_ release in bacterial RNAP is faster than that in Pol II [25]. This faster dynamics is due to several factors: First, the secondary channel of RNAP is shorter than the that of Pol II due to the absence of the funnel region, therefore the (Mg-PP_i_)^2−^ release path is shorter, which leads to a faster PP_i_ release from the active site of RNAP. Next, in RNAP, the PP_i_ only needs to overcome a single free energy barrier before it can be released to the solvent (See Figure 6A). In contrast, the PP_i_ release in Pol II was found to go over multiple free energy barriers sequentially before it could be released (See Figure 6B). Furthermore, in our model for RNAP, the S2 state (in the pore) has a population over seven times larger than that of the S1 state (in the active site). Thermodynamically, this difference will favor release of PP_i_ from the active site. However, in Pol II, the equilibrium population of the S2 state (the first state in the pore) is comparable to that of the S1 state (in the active site) [25]. This difference may be due to the fact that the S2 state in bacterial RNAP is greatly stabilized by four positively charged residues that are spatially close to each other (K908, K912, K780 and K1369), but in Pol II, these positively charged residues locate at relatively separate sites (See Figure 1A). Finally, R1239 in RNAP can substantially facilitate the (Mg-PP_i_)^2−^ release from the active site all the way to the solvent due to its longer and more flexible side chain (See Figure 5). However, its counterpart residue for PP_i_ release from Pol II, H1085, only promotes the PP_i_ escape from the active site to the first metastable state, S1, rather than all the way to solvent [25].

## Methods

We constructed the MSM to study the PP_i_ release in RNAP, and our algorithm consists of the following steps: (1) Model the PP_i_ bound complex. (2) Generate initial release pathways using SMD simulations. (3) Seed unbiased MD simulations from these initial pathways, and (4) Construct the MSM to identify metastable intermediate states and obtain both thermodynamics and kinetics of the PP_i_ release.

### 1. System setup and MD simulations

#### Setup of the bacterial RNAP elongation complex (EC)

The RNAP model was built from the crystal structure of the *T. thermophilus* RNAP bound with a non-hydrolysable substrate analogue, AMPCPP (PDB ID: 2O5J) [6]. Two missing motifs in the β′ subunit, from the residues 208 to 390 and from 1272 to 1328, were replaced with three GAG residues. Since these two motifs are largely exposed to the solvent, we thought they would not affect the dynamics of the PP_i_ release. Other parts of the EC, including four subunits (β, α1, α2 and ω domains), the downstream DNA, DNA-RNA hybrid, two Mg^2+^ ions, two Zn^2+^ ions, five crystal waters in the active site and the AMPCPP molecule, were retained.

#### Modeling the ATP- and PP_i_- bound RNAP complexes

We created ATP-bound RNAP complex by replacing the bridge carbon atom that connects the P_α_ and P_β_ atoms of AMPCPP with an oxygen atom. The minimized ATP-bound RNAP complex exhibits reasonable deviations from the crystal structure (See SI Figure S1A). Based on this ATP-bound RNAP complex, we built the PP_i_-bound RNAP complex by cleaving the P_α_-O bond of the ATP to form the PP_i_ group product.

The AMBER03 force field [42] was used to describe the protein residues, DNA, RNA, and metal ions. The parameters of ATP were taken from our previous study [43]. The (Mg-PP_i_)^2−^ group was treated as one group due to the significant internal charge transfer and its parameters were adopted from our previous study [25].

#### Molecular Dynamics (MD) simulation details

We used GROMACS 4.5 to conduct all the MD simulations [44]. Each EC was solvated with SPC water [45] in a cubic box and the minimum distance from the protein to the wall was 7.0 Å. To neutralize the system, 77 Na^+^ ions were added. There are 297,944 atoms in the final PP_i_ bound RNAP complex. Van der Waals and short-range electrostatic interactions were cut off at 10 Å. Long-range electrostatic interactions were treated with the Particle-Mesh Ewald (PME) summation method [46], [47]. The MD simulations were run at 1 bar and 310K using the Berendsen barostat [48] and the velocity rescaling thermostat [49], respectively. The LINCS algorithm was used to constrain all the chemical bonds [50]. The time-step was 2 fs and we updated the neighbor list every 10 steps. The solvated system was minimized with the steepest decent minimization method followed by a 120 ps MD simulation with position restrains on the heavy atoms of the proteins, DNA and RNA chains. The minimized PP_i_-bound RNAP complex displays minor fluctuations for the active site residues comparing to the crystal structure (See SI Figure S1B), indicating that our model is a good starting point.

### 2. Generating initial PP_i_ release pathways using SMD

In order to obtain the initial PP_i_ release pathway, we applied steered MD simulations [51] to pull the (Mg-PP_i_)^2−^ group out of the active site. The pulling was performed along three directions with the aim of considering all the possible PP_i_ release pathways. Three groups of residues were used to determine the pulling directions: β′ subunit residues 1136–1145, 908–914 and 1246–1253 (named as group I, II and III respectively). Two sets of pulling simulations were along the wall of the secondary channel: one was pulled towards the center of the C_α_ atoms of group I residues, and the other was directed to the center of C_α_atoms of both group I and group II residues. The third set of pulling simulations pointed to the center of the C_α_atoms of group II and group III residues, and toward the solvent. The external force was only applied on the center of mass of the PP_i_ group with a force constant of 0.5 kJ mol^−1^ Å^−2^ and pulling rate of 0.01 Å/ps. For each pulling direction, five independent steered MD simulations were performed starting from the final snapshot derived from 5 parallel MD simulations of the PP_i_-bound RNAP complex.

### 3. Seeding unbiased MD simulations

We first divided the conformations from SMD simulations into 20 clusters using the K-center clustering algorithm [52]. In the clustering, the distance between a pair of conformations was set to be the RMSD value of three PP_i_ atoms (the bridge oxygen and two phosphate atoms). To compute RMSD, the structure was aligned to the energy minimized PP_i_-RNAP complex by the C_α_atoms of the bridge helix domain. We then randomly selected 5 conformations from each cluster (a total of 100 conformations) to conduct unbiased MD simulations. Each simulation was run for 10 ns and the snapshots were saved every 2 ps. Altogether, we obtained an aggregation of ∼1 µs simulations with 500,000 conformations.

### 4. Constructing the Markov State Model (MSM)

In MSM, the conformational space is divided into a number of metastable macrostates and the fast motions are integrated out by coarse graining in time with a discrete unit of *Δt*. The model is markovian if *Δt* is longer than the intra-state relaxation time. In other words, the probability for the system to be at a given state at time *t+Δt* only depends on the state at time *t*. In MSM, the long timescale dynamics can be modeled by the first-order master equation.
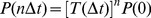
(1)Where *P*(*nΔt*)is the state populations vector at time *nΔt*, and *T* is the transition probability matrix. *Δt*is the lag time of the model. To calculate *T*, one can normalize the transition count matrix generated by counting the number of transitions between each pair of states at the observation interval of *Δt* from MD trajectories. MSM has been successfully applied to model conformational changes that occur at timescales that cannot be directly accessed by conventional MD simulations such as protein folding [39], [40], [53]–[55].

To construct the MSM, we have followed a splitting and lumping procedure [52]:

#### Splitting MD conformations into microstates

We have divided all the conformations from our seeding MD simulations (500,000 conformations) into 200 microstates by employing the K-center clustering algorithm [52]. The distance between a pair of conformations was set to be the RMSD value of three PP_i_ atoms (the bridge oxygen and two phosphate atoms). To compute RMSD, the structure was aligned to the modeled PP_i_-bound RNAP complex according to the C_α_ atoms of the BH residues. The microstates are small, and the average RMSD values to its central conformation in each state are only ∼2 Å.

The transition probability matrix *T_ij_* from state *i* to state *j* was obtained by counting the transition numbers *N_ij_* observed in the MD trajectories within a certain lag time Τ, and normalizing each row by: 
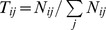
We used the sliding-window to count the transitions due to the limited samplings. To avoid the re-crossing events, we count the transitions from *i* to *j* only if the state *j* can stay in *j* at least within 50 ps without transferring to other states.

#### Lumping microstate into macrostates

We applied the Robust Perron Cluster Cluster Analysis (PCCA+) algorithm [56] to lump the 200 microstates obtained above into 2 macrostates. The number of macrostates was determined from the implied timescale plot of the 200 microstate. The plot levels off starting from the lag time of 4 ns and exhibits one clear gap, suggesting that two macrostates exist (See SI Figure S3A). Finally we chose a lag time of 4.5 ns to calculate the equilibrium populations and the Mean First Passage Time (MFPT).

We calculated the MFPTs to estimate the average transition rates between each macrostates pair. As described before [25], [54], the MFPT can be determined using the following formula: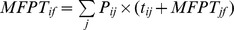 where *P_ij_* is the transition probability from state *i* to state *j*, *t_ij_* is the lag time used to construct the transition probability matrix ***T**_ij_*, *t_ij_* is equal to 4.5 ns in our model, and MFPT*_if_* is the mean first passage time of the state *j* to final state *f*. For each transition, a set of linear equations can be solved under the boundary condition in which MFPT_*ff*_ = 0. The uncertainties of the MFPT were obtained by bootstrapping the MD trajectories for 100 times.

### 5. Validating the Markov State Model

In order to check if the model is markovian, we have plotted the implied timescales (*τ_k_*) as a function of the lag time *τ*:
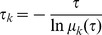
(2)where *μ_k_* is the eigenvalue of the transition probability matrix *T* with the lag time *τ*. The implied timescales correspond to the average transition times between two groups of states, and thus indicate the dynamics of the system. If *τ* is sufficiently large, the model is markovian, and the predicted implied timescales will not change upon the further increase of the lag time. In our system, the implied timescale plots reach the plateau at the lag time of ∼4 ns (See SI Figure S3A). Therefore, we select the lag time of 4.5 ns to construct the final MSM.

To further validate the model, we predicted the probability for a given macrostate to stay within it after a certain lag-time based on our MSM, and this predicted values are in good agreement with those obtained from the original MD simulations (See SI Figure S3B).

### 6. Control simulations of the truncated systems

In order to investigate the stability of the TL in free solution, we have performed a 300 ns control simulation with the isolated TL domain (A1225 to A1265) in solution (with ∼6900 atoms, See SI Figure S6B). However, it is difficult to extend individual MD simulations of the complete transcription complex (nearly 300 K atoms) to hundreds of nanoseconds due to its high computing cost. Therefore, we have also performed simulations with a truncated RNAP complex containing all the motifs surrounding the TL domain (See SI Figure S7A), including β subunit residues 381–569, 831–1049, β′ residues 604–794, 901–1470, 10 upstream hybrid DNA-RNA base pairs, 6 downstream DNA base pairs and Mg^2+^A in the active site. The final solvated system only contains ∼118 K atoms, but it still takes more than 2 months to perform one 300-ns simulation using 24 CPU cores.

The explicit SPC water model was used for the MD simulations, 1 and 29 Na^+^ ions were added to neutralize the isolated TL and truncated RNAP model respectively. The other setups for the MD simulations were the same as the seeding MD simulations. We have performed one 300-ns simulation for the isolated TL and the other two 300-ns simulation for the truncated RNAP. For the truncated RNAP model, several terminal residues that are truncated from the complete model were fixed in the simulations in order to avoid undesired unfolding.

## Supporting Information

Figure S1 Energy minimized structures of the ATP-bound (A) and PP_i_-bound RNAP complexes (B). The two complexes are superimposed with the crystal structure of the AMPCPP bound RNAP complex (PDB ID: 2O5J, gray). The BH, TL, RNA chain, Mg^2+^ ions and the the substrate are shown in green, magenta, red, yellow, and organe/red, respecitively. Several residues around the active site are also highlighted.
(TIF)

Figure S2 Our 100 unbaised MD simulations widely sampled the secondary channel. Three conformations from each simulation at 0 ns, 5 ns and 10 ns are shown as spheres, and connected by sticks.
(TIF)

Figure S3 (A). Implied timescale plot as the function of the lag time for the 200-microstates MSM (left panel) and 2-macrostates MSM (right panel). (B). Validation of our MSM. The probability for a given macrostate to stay within it after a certain lag-time can be predicted from our MSM (blue dashed lines), and this predicted values are comparable to the direct counts from the MD simulations (red dashed lines). The lag time we used is 4.5 ns.
(TIF)

Figure S4 (A) The distance between the PP_i_ group and the P_α_ as the function of the simulation time for R1029K mutant MD simulation initiated from P1 conformation. (B) R1029K mutant MD simulations initiated from P2, the distance between PP_i_ group and Mg^2+^A was shown. (C) Same as (A) but for R1029K mutant MD simulation. Please refer to the caption of Figure 4A for additional details.
(TIF)

Figure S5 Potential of mean force (PMF) plots for: (A) the complete TL (Q1235 to G1255) and (B) the TL tip (R1239 to A1249). Both of the PMF plots were projected on two reaction coordinates: the distance between the PP_i_ group and the Mg^2+^A (d_1_), and the heavy-atom RMSD comparing to the energy minimized PP_i_-bound RNAP complex.
(TIF)

Figure S6 MD simulation of the isolated TL in solution. (A) The heavy-atom RMSD of the TL residues (from Q1255 to G1275) as a function of the simulation time. (B) Structures of two snapshots from the MD simulation at 0ns and 300ns. (C) The secondary structure analysis of the TL domain along the simulation time.
(TIF)

Figure S7 MD simulations of the truncated RNAP complex. (A) The initial structure of the truncated RNAP complex. (B) The heavy-atom RMSD of the TL residues (from Q1255 to G1275) as a function of the simulation time for two independent MD simulations. (C) The secondary structure analysis of the TL domain along the simulation time.
(TIF)

Table S1 Mean First Passage Time (MFPT) obtained from our MSMs for transitions between two metastable states. See Methods section for details of the MFPT calculations.
(DOCX)
