# Supplementary figures and images for: A Two-State Model for the Dynamics of the Pyrophosphate Ion Release in Bacterial RNA Polymerase
Source: PLoS Comput Biol. 2013 Apr 4;9(4):e1003020. doi: 10.1371/journal.pcbi.1003020 (PMC3617016; doi:10.1371/journal.pcbi.1003020)

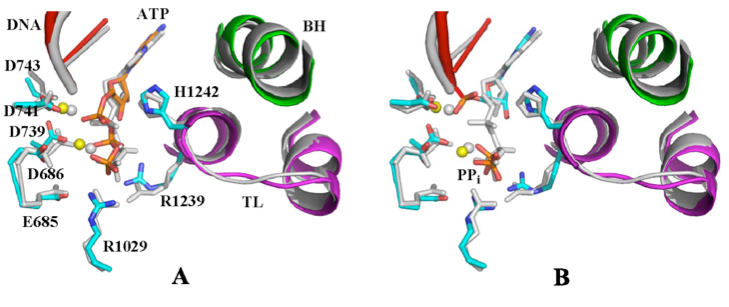

Supplement: Figure S1 — Energy minimized structures of the ATP-bound (A) and PPi-bound RNAP complexes (B). The two complexes are superimposed with the crystal structure of the AMPCPP bound RNAP complex (PDB ID: 2O5J, gray). The BH, TL, RNA chain, Mg2+ ions and the the substrate are shown in green, magenta, red, yellow, and organe/red, respecitively. Several residues around the active site are also highlighted. (TIF) [file pcbi.1003020.s001.tif]

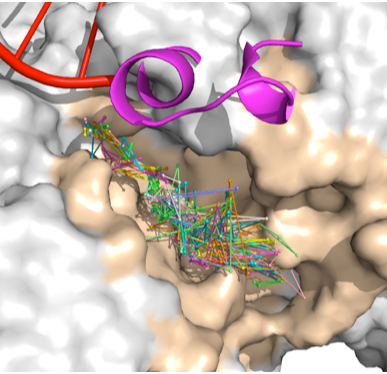

Supplement: Figure S2 — Our 100 unbaised MD simulations widely sampled the secondary channel. Three conformations from each simulation at 0 ns, 5 ns and 10 ns are shown as spheres, and connected by sticks. (TIF) [file pcbi.1003020.s002.tif]

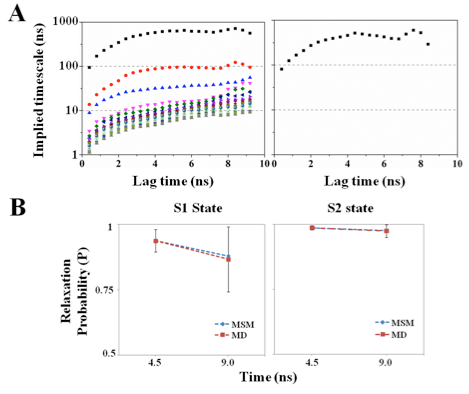

Supplement: Figure S3 — (A). Implied timescale plot as the function of the lag time for the 200-microstates MSM (left panel) and 2-macrostates MSM (right panel). (B). Validation of our MSM. The probability for a given macrostate to stay within it after a certain lag-time can be predicted from our MSM (blue dashed lines), and this predicted values are comparable to the direct counts from the MD simulations (red dashed lines). The lag time we used is 4.5 ns. (TIF) [file pcbi.1003020.s003.tif]

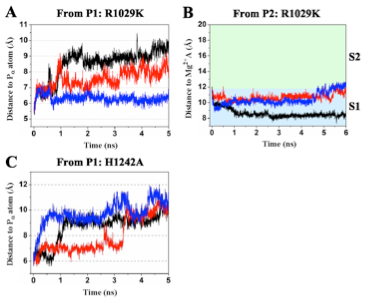

Supplement: Figure S4 — (A) The distance between the PPi group and the Pα as the function of the simulation time for R1029K mutant MD simulation initiated from P1 conformation. (B) R1029K mutant MD simulations initiated from P2, the distance between PPi group and Mg2+A was shown. (C) Same as (A) but for R1029K mutant MD simulation. Please refer to the caption of Figure 4A for additional details. (TIF) [file pcbi.1003020.s004.tif]

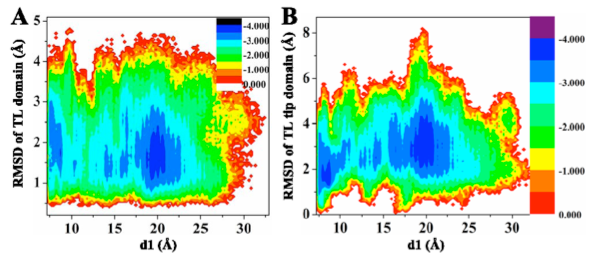

Supplement: Figure S5 — Potential of mean force (PMF) plots for: (A) the complete TL (Q1235 to G1255) and (B) the TL tip (R1239 to A1249). Both of the PMF plots were projected on two reaction coordinates: the distance between the PPi group and the Mg2+A (d1), and the heavy-atom RMSD comparing to the energy minimized PPi-bound RNAP complex. (TIF) [file pcbi.1003020.s005.tif]

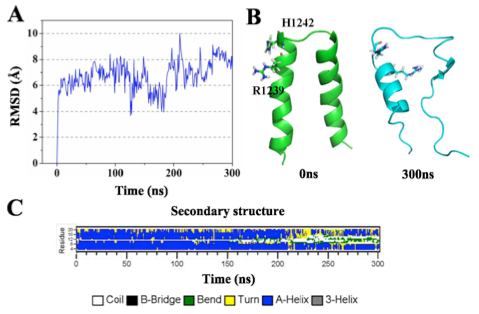

Supplement: Figure S6 — MD simulation of the isolated TL in solution. (A) The heavy-atom RMSD of the TL residues (from Q1255 to G1275) as a function of the simulation time. (B) Structures of two snapshots from the MD simulation at 0ns and 300ns. (C) The secondary structure analysis of the TL domain along the simulation time. (TIF) [file pcbi.1003020.s006.tif]

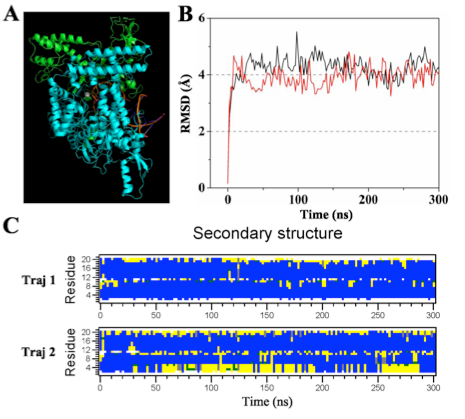

Supplement: Figure S7 — MD simulations of the truncated RNAP complex. (A) The initial structure of the truncated RNAP complex. (B) The heavy-atom RMSD of the TL residues (from Q1255 to G1275) as a function of the simulation time for two independent MD simulations. (C) The secondary structure analysis of the TL domain along the simulation time. (TIF) [file pcbi.1003020.s007.tif]
