# Supplementary material for: A Two-State Model for the Dynamics of the Pyrophosphate Ion Release in Bacterial RNA Polymerase
Source: PLoS Comput Biol. 2013 Apr 4;9(4):e1003020. doi: 10.1371/journal.pcbi.1003020 (PMC3617016; doi:10.1371/journal.pcbi.1003020)
Supplement: Table S1 — Mean First Passage Time (MFPT) obtained from our MSMs for transitions between two metastable states. See Methods section for details of the MFPT calculations. (DOCX) [file pcbi.1003020.s008.docx]

**Table S1**. Mean First Passage Time (MFPT) obtained from our MSMs for transitions between two metastable states. See Methods section for details of the MFPT calculations.

|  | S1 to S2 | S2 to S1 |
| --- | --- | --- |
| MFPT (ns) | 556±150 | 2574±694 |
